# Correction: Type B and type A influenza polymerases have evolved distinct binding interfaces to recruit the RNA polymerase II CTD

**DOI:** 10.1371/journal.ppat.1011073

**Published:** 2023-01-04

**Authors:** Tim Krischuns, Catherine Isel, Petra Drncova, Maria Lukarska, Alexander Pflug, Sylvain Paisant, Vincent Navratil, Stephen Cusack, Nadia Naffakh

The Acknowledgments section is incomplete. The full Acknowledgments section should read as follows:

We thank Pr. Daniel Perez (University of Georgia), Dr. Bernard Delmas (INRAE) and Dr. Yves Janin (Institut Pasteur) for sharing material and reagents, and Dr. Yves Jacob for helpful discussions. We acknowledge the European Synchrotron Radiation Facility for provision of synchrotron radiation facilities and we would like to thank the staff of the ESRF and EMBL Grenoble for assistance and support in using beamlines ID29 and ID30A1 (MASSIF).
