# Supplementary figures and images for: Type B and type A influenza polymerases have evolved distinct binding interfaces to recruit the RNA polymerase II CTD
Source: PLoS Pathog. 2022 May 23;18(5):e1010328. doi: 10.1371/journal.ppat.1010328 (PMC9242477; doi:10.1371/journal.ppat.1010328)

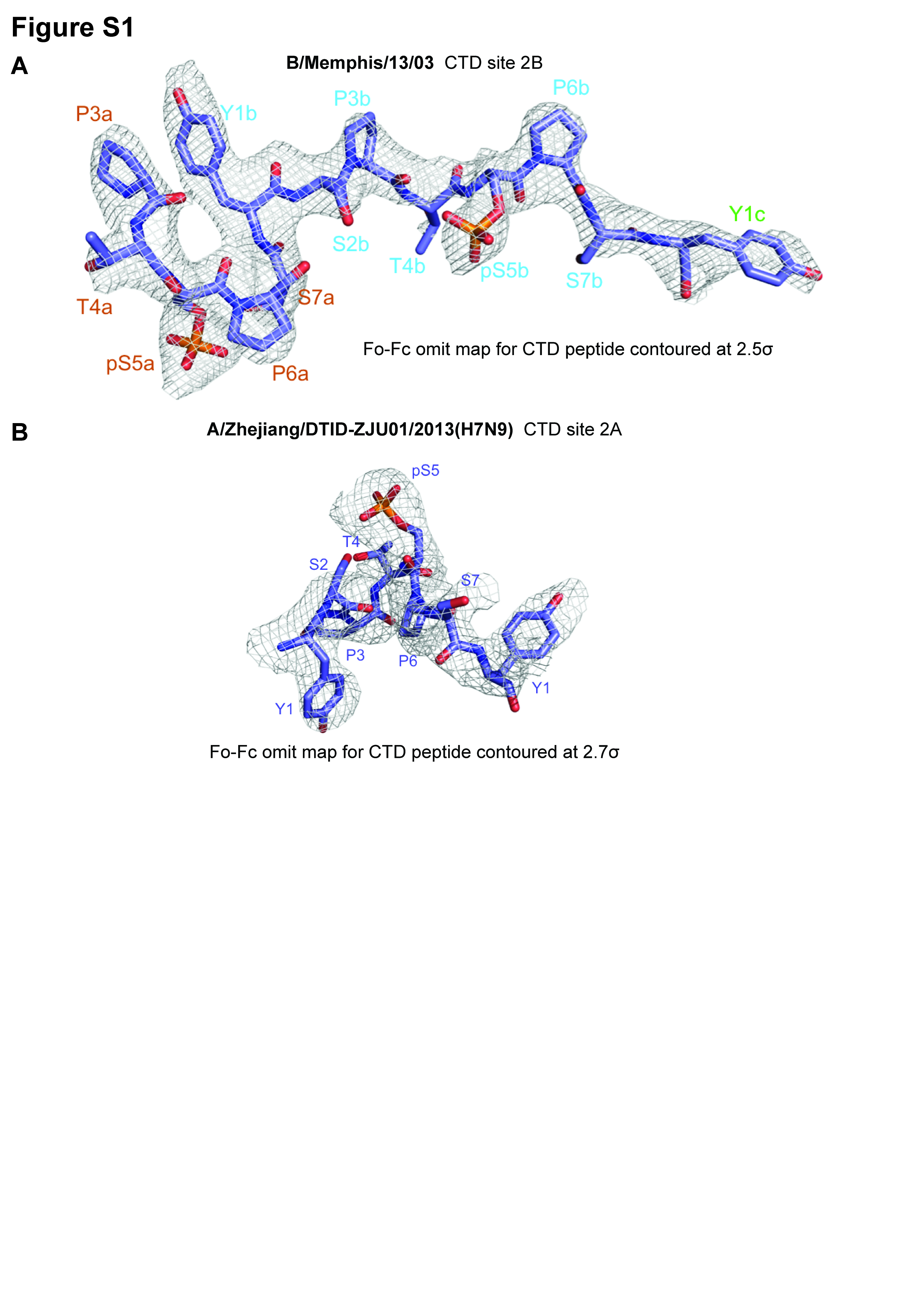

Supplement: S1 Fig — A. CTD peptide bound in site 2B of FluPolB (B/Memphis/13/2003) polymerase. Fo-Fc omit map shown at 2.5 σ with clear density for two phosphoserines (pS5a and pS5b). B. CTD peptide bound in site 2A of FluPolA (A/Zhejiang/DTID-ZJU01/2013(H7N9)) polymerase. Fo-Fc omit map shown at 2.7 σ with clear density for one phosphoserine. (TIF) [file ppat.1010328.s001.tif]

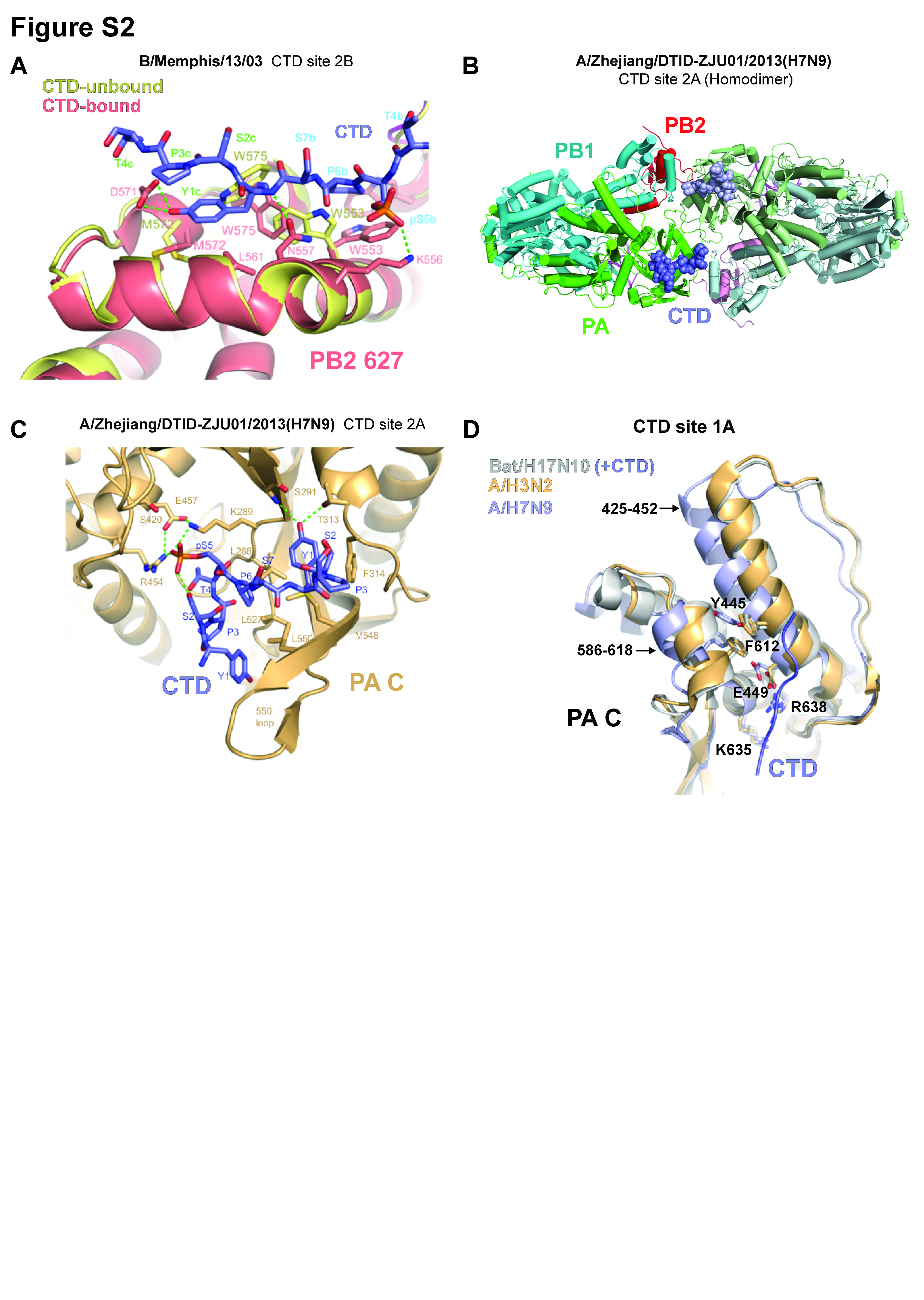

Supplement: S2 Fig — A. Superposition of the PB2-627 domains of CTD-bound (deep salmon with CTD in slate-blue) and unbound (wheat) FluPolB (B/Memphis/13/2003) polymerase, showing induced-fit conformational changes of three key hydrophobic residues (PB2 W553, M572, W575). B. Ribbon diagram looking down the 2-fold axis of the symmetrical homodimeric A/H7N9 polymerase core with CTD peptide bound in site 2A. One monomer is coloured with PA (green), PB1 (cyan), PB2 (red) with CTD peptide (slate spheres) and 5’ vRNA hook (violet). The second monomer has paler versions of these colours. C. Details of the binding of the CTD peptide (slate blue) in site 2A of FluPolA (A/Zhejiang/DTID-ZJU01/2013(H7N9) core) polymerase. D. Comparison of site 1A configuration for CTD bound form of FluPolA (bat influenza A (A/little yellow-shouldered bat/Guatemala/060/2010(H17N10) [32], PDB: 5M3H, PA subunit light grey, CTD peptide slate-blue), CTD free, transcription active form of FluPolA (A/NT/60/1968 (H3N2), [58], PDB: 6RR7, wheat) and dimeric FluPolA (A/Zhejiang/DTID-ZJU01/2013(H7N9) core, light blue, this work). The FluPolA (H7N9) polymerase core is the symmetrical dimer with each polymerase in the open, ‘dislocated’ state [18]. Due to the dislocation, PA regions 425–452 and 586–618 are rotated by ~20°, which particularly effects the position of site 1A binding site residues Y445, E449 and F612. This likely explains the lack of CTD binding observed in site 1A for the H7N9 core, whereas site 2A is undistorted and occupied by CTD (S2B Fig). (TIF) [file ppat.1010328.s002.tif]

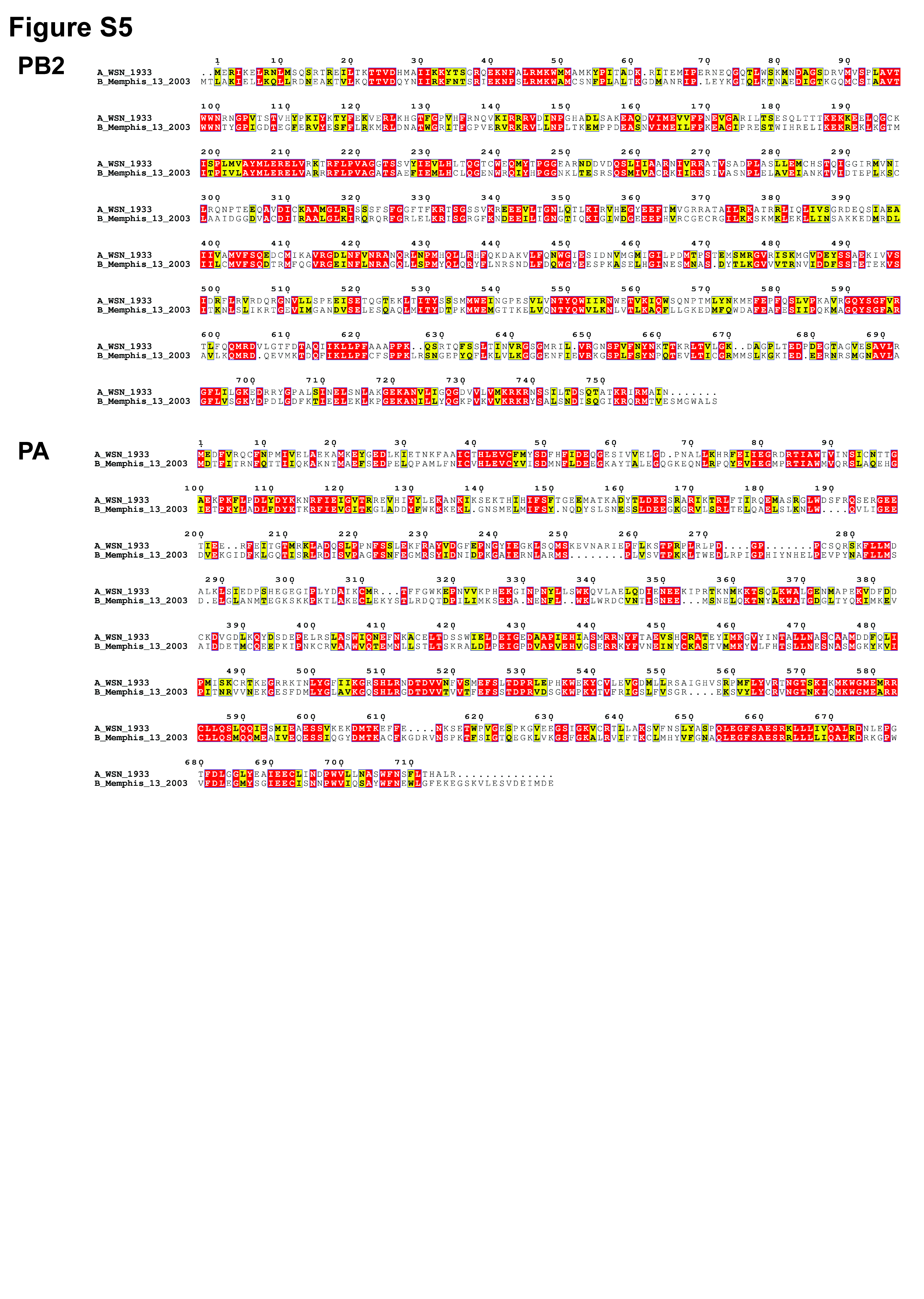

Supplement: S5 Fig — Protein sequences were obtained from UniProt (https://www.uniprot.org/), aligned with SnapGene 6.0 and visualized by Espript 3.0 [55]. Identical and similar residues are indicated in red or yellow, respectively. (TIF) [file ppat.1010328.s005.tif]

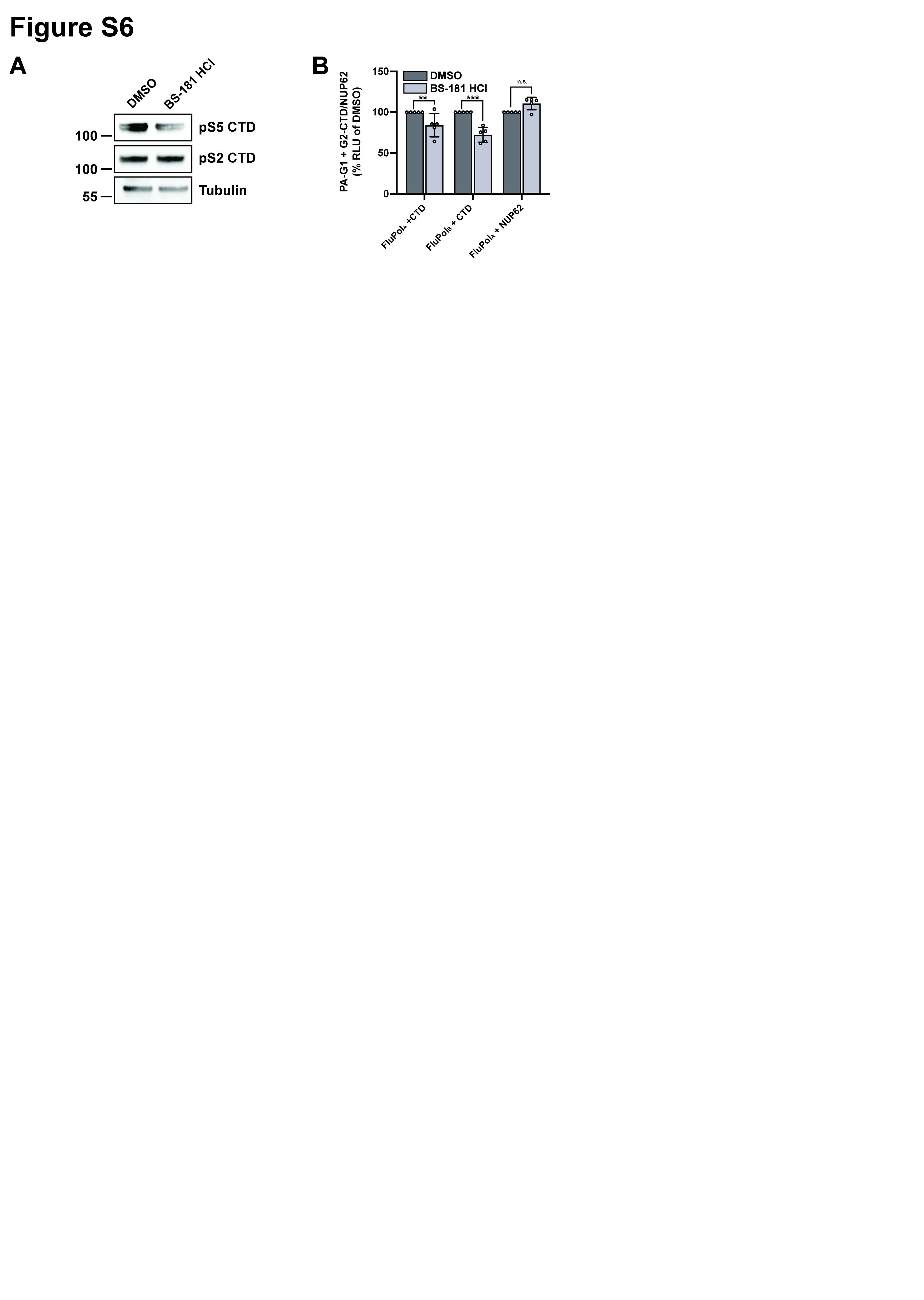

Supplement: S6 Fig — A. HEK-293T cells were transfected with G2-CTD. At 24 hpt cells were treated for 1 h with 20 μM BS-181-HCl (DMSO final concentration 0.2%). Cell lysates were analysed by western blot with antibodies specific for pS5 or pS2 CTD and tubulin. B. In vivo CTD binding of FluPolA (A/WSN/1933) and FluPolB (B/Memphis/13/2003). G2-tagged CTD was expressed by transient transfection in HEK-293T cells together with the viral polymerase subunit PB2, PB1 and PA-G1. At 24 hpt cells were treated for 1 h with 20 μM BS-181-HCl or 0.2% DMSO before cell lysis and measurement of G. princeps luciferase activity as described in the Materials and Methods section. As a control, the previously described FluPolA−NUP62 interaction was investigated by co-transfection of G2-NUP62, PB2, PB1 and PA-G1. RLUs are expressed as percentages relative to DMSO treated cells. The data shown are mean ± SD of five independent experiments performed in technical triplicates. ***p ≤ 0.002, ***p ≤ 0.001 (two-way ANOVA; Dunnett’s multiple comparisons test). (TIF) [file ppat.1010328.s006.tif]

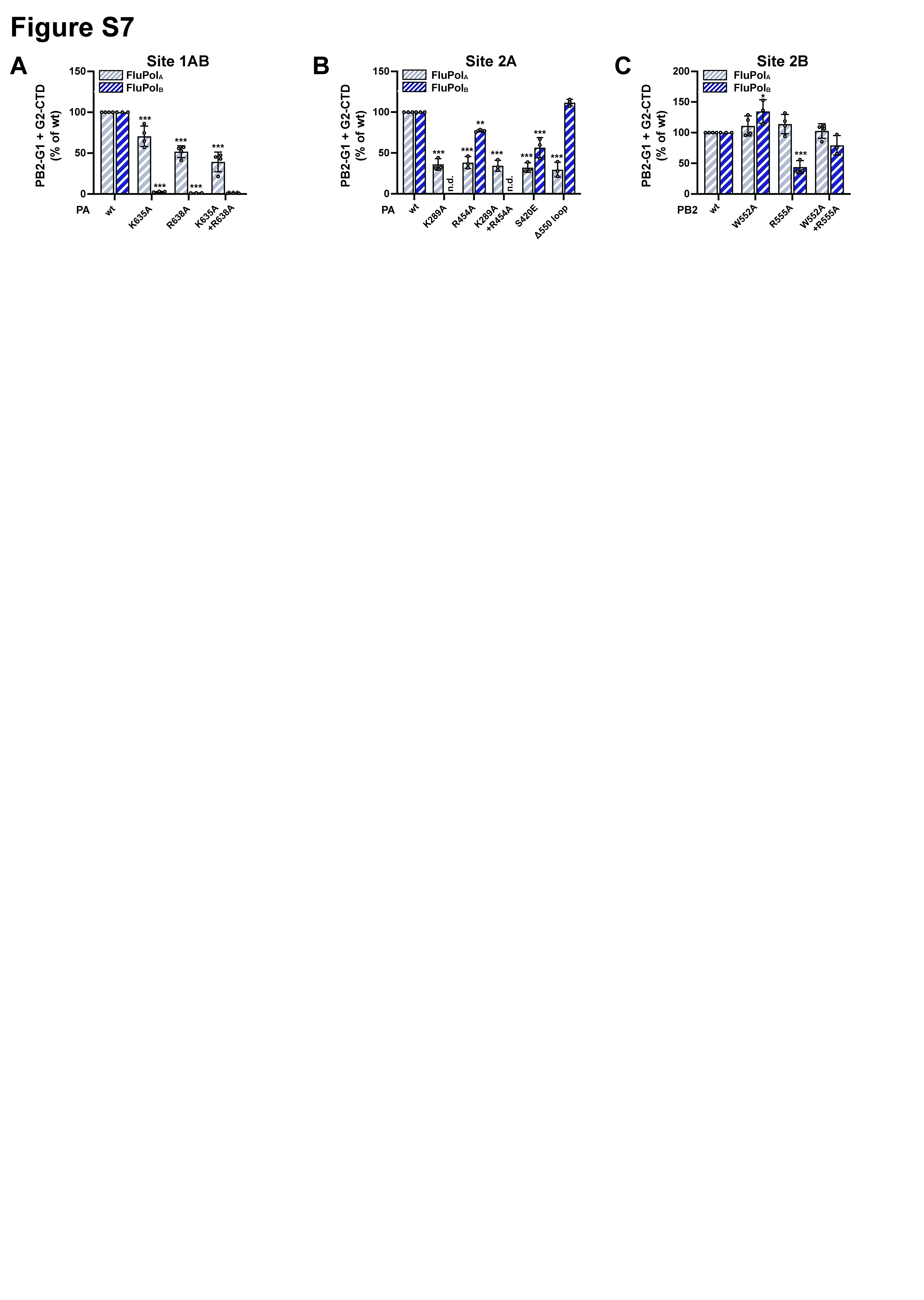

Supplement: S7 Fig — A-C. In vivo CTD binding of the indicated site 1AB (A), site 2A (B) and site 2B (C) mutants of FluPolA (A/WSN/1933, grey hatched bars) and FluPolB (B/Memphis/13/2003, blue hatched bars). The G2-tagged CTD was expressed by transient transfection in HEK-293T cells together with PB2-G1, PB1 and PA. RLUs are expressed as percentages relative to wt FluPolA/B. The data shown are the mean ± SD of at least three independent experiments performed in technical triplicates. **p ≤ 0.002, ***p ≤ 0.001 (two-way ANOVA; Dunnett’s multiple comparisons test). (n.d.) not determined. (TIF) [file ppat.1010328.s007.tif]

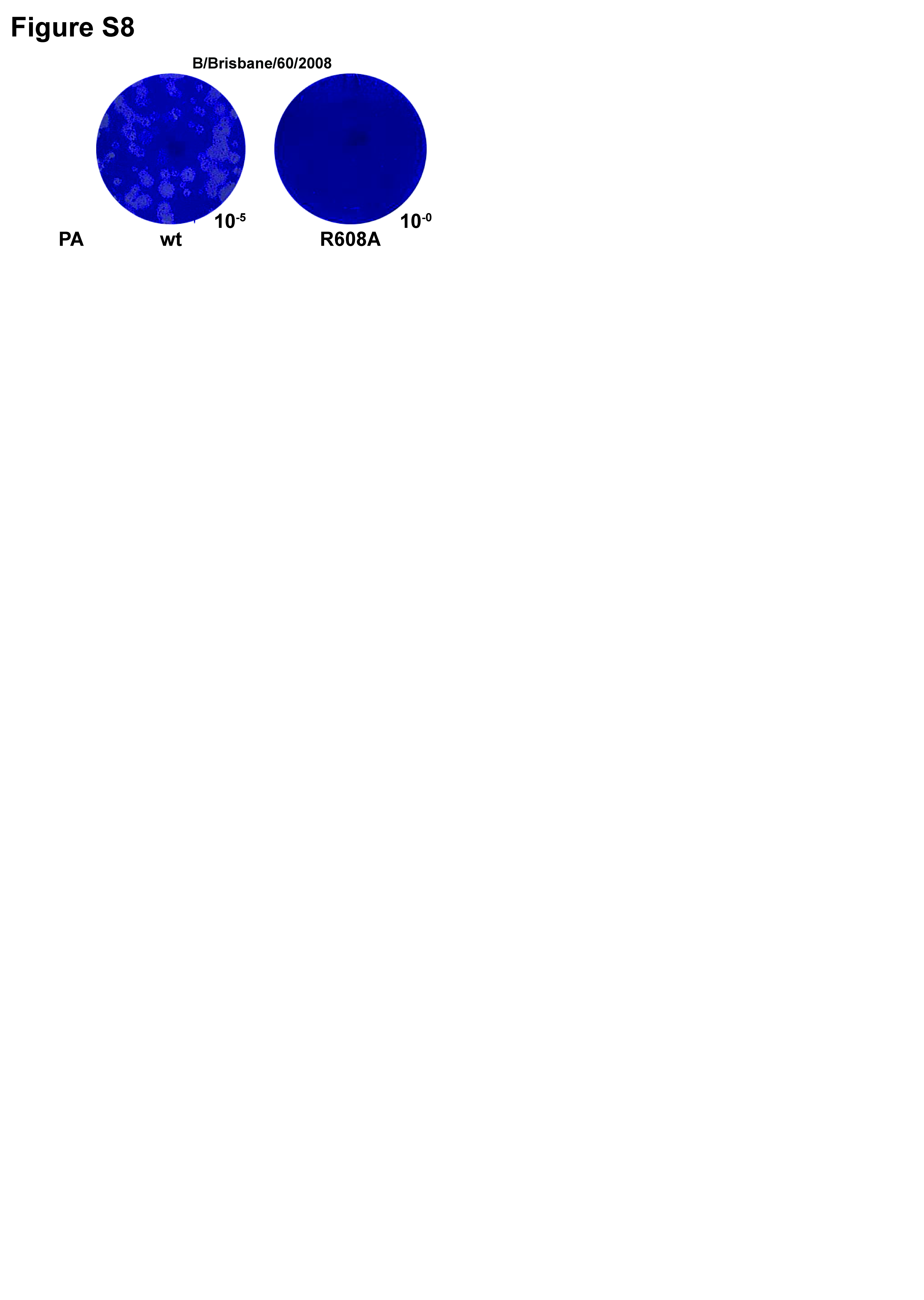

Supplement: S8 Fig — Characterisation of recombinant IBV (B/Brisbane/60/2008) PA R608A mutant virus. Recombinant viruses with the indicated mutations were generated by reverse genetics as described in the Materials and Methods section. Reverse genetic supernatants were titrated on MDCK cells and stained at 72 hpi by crystal violet. The pictures show one representative plaque assay with the indicated ten-fold dilution. (TIF) [file ppat.1010328.s008.tif]

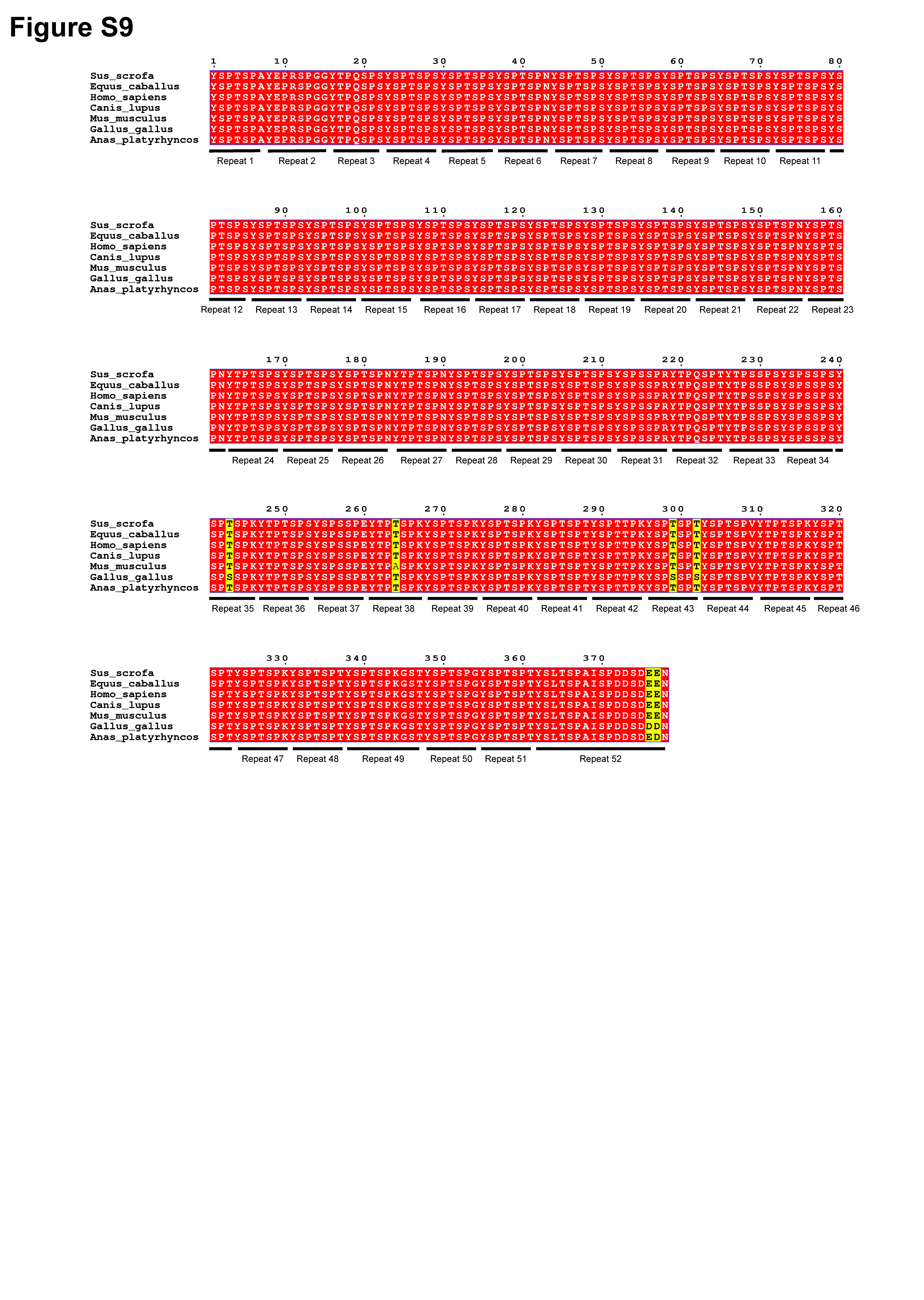

Supplement: S9 Fig — The RPB1 CTD sequences of Sus scrofa (wild boar), Equus caballus (horse), Homo sapiens (human), Canis lupus (wolf), Mus musculus (house mouse), Gallus gallus (chicken) and Anas platyrhynchos (wild duck) were obtained as described in the Materials and Methods section, aligned with SnapGene 6.0 and visualised by Espript 3.0 [55]. The CTD repeat numbers are indicated below the sequence alignment. Identical and similar residues are indicated in red or yellow, respectively. (TIF) [file ppat.1010328.s009.tif]
